# A phase 2b basket trial approach to treat multiple rare and fibrotic skin diseases

**DOI:** 10.3389/fmed.2025.1637040

**Published:** 2025-09-23

**Authors:** Sebastian Volc, Peter Martus, Matthias Schefzyk, Claudia Günther, Pia Moinzadeh, Laura Susok, Rubén A. Ferrer, Manola Zago, Christiane Pfeiffer

**Affiliations:** ^1^Department of Dermatology, Eberhard Karls University, Tuebingen, Germany; ^2^Institute for Clinical Epidemiology and Applied Biostatistics, Eberhard Karls University, Tuebingen, Germany; ^3^Department of Dermatology, Allergology und Venerology, Hannover Medical School, Hannover, Germany; ^4^Department of Dermatology, TU Dresden, Dresden, Germany; ^5^Department of Dermatology and Venereology, University Hospital Cologne, Cologne, Germany; ^6^Department of Dermatology, Klinikum Dortmund gGmbH, University Witten/Herdecke, Dortmund, Germany; ^7^Department of Dermatology and Allergy, University Hospital, LMU Munich, Munich, Germany; ^8^Center for Clinical Trials, University Hospital of Tuebingen, Tuebingen, Germany

**Keywords:** rare diseases, basket trial, Simon design, fibrotic skin diseases, clinical trial design, clinical score

## Abstract

Fibrotic skin diseases are rare, chronic, and often debilitating conditions characterized by excessive extracellular matrix deposition, leading to tissue scarring and functional impairment. Despite their severity, diseases—such as lichen sclerosus et atrophicus (LSA), frontal fibrosing alopecia (FFA), radiation-induced skin fibrosis (RISF), eosinophilic fasciitis (EF), pansclerotic disabling morphea (PDM), and linear circumscript sclerodermia (LCS)—lack approved therapies and are underrepresented in clinical research. This phase 2b multicenter basket trial proposes a novel approach to evaluate a common antifibrotic therapy across these diverse but pathophysiologically related conditions. The trial employs a two-stage Simon design to address the statistical challenges posed by small patient populations, allowing the inclusion of ultra-rare diseases while maintaining analytical rigor. LSA and FFA serve as primary study groups due to higher prevalence, while EF, RISF, PDM, and LCS are included as exploratory arms. The study aims to assess the efficacy, safety, and tolerability of the selected therapy, while also providing mechanistic insights into fibrosis through molecular analyses. The primary endpoint is a ≥ 1-point improvement in the Investigator Global Assessment (IGA) at 24 weeks. Secondary endpoints at 52 weeks encompass quality of life (Dermatological Life Quality Index (DLQI), EuroQol Group Quality of Life Questionnaire (EuroQol five dimensions (EQ-5D))), symptom relief (itch and pain Numeric Rating Scale (NRS)), and disease-specific clinical scores. The trial excludes a placebo arm due to ethical considerations in progressive, untreated diseases but allows rescue therapies for disease progression. This design not only facilitates access to treatment for underserved populations but also leverages shared clinical and molecular features to enhance statistical power. By integrating disease-specific and global outcome measures, the study aims to generate robust evidence for repurposing existing therapies. If successful, this trial could serve as a model for future research in rare fibrotic diseases, accelerating drug development and improving patient outcomes.

## 1 Introduction

Fibrotic diseases are characterized by excessive deposition of extracellular matrix components, leading to tissue scarring and organ dysfunction. Many fibrotic diseases are non-monogenetic rare diseases affecting less than 1 in 10,000 people and pose difficulties for targeted drug development.

In fibrotic skin diseases, such as, but not limited to:

- Extragenital and genital lichen sclerosus et atrophicus (LSA).
- Frontal fibrosing alopecia (FFA).
- Radiation-induced skin fibrosis (RISF).
- Eosinophilic fasciitis (EF).
- Pansclerotic disabling morphea (PDM).
- Linear circumscript sclerodermia (LCS).

There is an unmet therapeutic need, as no approved treatment options are available, and trials with novel treatments are scarce or non-existent. Guidelines are based on steroid therapy (topical and/or systemic), topical calcineurin inhibitors, methotrexate, and phototherapy, which are employed to various degrees of efficacy. All of these diseases are physically debilitating and may even be painful. They confer psychological burdens, such as shame and stigmatization, encompassing a downward spiral to social withdrawal, loneliness, and depression (1–3). Furthermore, they may present in childhood and adolescence with a lasting psychological impact during those formative years (4, 5). A clinical trial studying a new drug targeting a key pathophysiological event in those fibrosing diseases should therefore focus on intervening in the early inflammatory stages of the diseases in children and adults, employing a design that allows overcoming the restrictions given by the low number of affected individuals and offering a possibility to reach a clinically and statistically relevant endpoint.

Fibrotic diseases are characterized by the excessive deposition of extracellular matrix components, leading to tissue scarring and organ dysfunction. Among the cytokines implicated in fibrosis, interleukin 13 (IL-13) has emerged as a critical mediator (6). IL-13 is secreted by Th2, NKT, and mast cells. It acts on monocytes, epithelial cells, B cells, and fibroblasts. Through fibroblast stimulation, it plays a role in promoting collagen synthesis, leading to tissue remodeling and fibrosis in the skin as well as in other organs, as in systemic sclerosis (SSc), hepatic fibrosis, idiopathic pulmonary fibrosis (IPF), asthma, and eosinophilic esophagitis. While inhibiting IL-4/−13 pathways was efficient in abrogating lung fibrosis in mouse models (7), phase 2 clinical trials in IPF, as well as in SSc with different antibodies, inhibiting IL-4 and IL-13 (8), or IL-13 with higher affinity (9), have not met the relevant endpoint (difference in decline in FVC), while skin fibrosis improved (9). Consequently, we aim to address several skin diseases, in which early events of fibrosis may be more detectable and thus allow a proof of principle of this approach, while establishing a base for quantitative assessment of the effect across several indications.

Therefore, we propose a basket trial for these fibrotic skin diseases with an IL-13-directed therapy.

Basket trials test how well a drug licensed for a limited number of indications works in patients with different types of diseases that share the same mutation, biomarker, or pathophysiological property (e.g., in oncological diseases with immune therapy), or in this case, the IL-13 signature. This design enables the simultaneous study of the drug’s effectiveness across multiple diseases. It may expedite the approval process for new treatments in general (10). Basket trials have been a common strategy in oncologic diseases for some time and have recently gained popularity in inflammatory diseases as well, allowing access to potentially beneficial treatments in a systematic trial design for otherwise untreated patients. Rare diseases are often characterized by a lack of licensed drugs based on the low number of affected subjects, limiting the power of trials aimed at developing or repurposing medications for their treatment. To improve the statistical power and enable the inclusion of rare diseases with very low clinical trial subject numbers, a statistical approach based on an adaptive 2-step Simon design will be employed (15, 16).

## 2 Methods

### 2.1 Objectives

The objectives of the study are:

1. To evaluate the efficacy, safety, and tolerability of IL-13 inhibition in reducing fibrosis in patients with LSA, FFA, RISF, EF, PDM, and LCS.
2. To understand the molecular mechanisms by which the therapeutic target contributes to fibrosis in these conditions.
3. To assess the safety and tolerability of the selected therapy in the clinical setting.

### 2.2 Expected outcomes

1. Quality of life improvement: Assessed during and after treatment with disease-specific scores as well as the Dermatological Life Quality Index (DLQI) in all patients and disease entities included (Table 1).
2. Reduction in fibrosis: Assessed with disease entity-specific imaging, histology, and clinical scoring: disease-specific scoring systems, as well as the Investigator Global Assessment (IGA), to evaluate the whole study population.
3. Mechanistic insights: Elucidate the pathways that drive fibrosis, providing a deeper understanding of disease pathogenesis.
4. Safety profile: Assessment of safety and tolerability profile for use of the selected therapy in the addressed disease entities, thereby expanding knowledge on the favorable safety profile observed in the already licensed indications.

**Table 1:** Entity-specific disease scores.

Disease entity	Entity specific scores	Global scores
LSA	LSCIS, CIV	IGA Itch NRS Pain NRS DLQI EQ-5D
FFA	FFASI, FFA-QoL	
EF	LoSCAT-A/D, mLoSSI, LoSDI	
RISF	LoSCAT-A/D	
PDM	LoSCAT-A/D	
LCS	LoSCAT-A/D	

CIV, Clitoral phimosis, Interlabial sulci involvement, Vulvar introitus narrowing; DLQI, Dermatological Life Quality Index; EQ-5D, EuroQol Group Quality of Life questionnaire; FFA-QoL, Frontal Fibrosing Alopecia Quality of Life; FFASI, Frontal Fibrosing Alopecia Severity Index; IGA, Investigator Global Assessment; LoSCAT-A/D, Localized Scleroderma Cutaneous Assessment Tool Activity/Damage; LoSDI, Localized Scleroderma Damage Index; LSCIS, Lichen Sclerosus Clinical Index of Severity; mLoSSI, modified Localized Scleroderma Skin Severity Index; NRS, Numeric Rating Scale.

To address the challenges in treatment development in rare and undertreated fibrotic skin diseases, we designed a multicenter phase 2b (proof of concept) trial employing a basket trial design.

Addressing fibrotic diseases in a basket trial, the trial design must be set up in a way that allows the inclusion of very rare diseases with *n* < 10 and still reach statistical significance. This can be achieved by grouping a multitude of entities together that share a common, hard endpoint (e.g., survival in oncological patients) or by establishing a statistical approach to allow for combining groups of uneven size within one statistical analysis. We chose the second approach to enable treatment for patients affected with rare diseases, while also treating patients suffering from diseases with higher incidence.

We designed a multicenter phase 2b (proof-of-concept) trial employing a basket trial design for two moderately large subcohorts (FFA *n* = 28; LSA *n* = 35) of patients. We chose optimal two-stage Simon designs in both cohorts combined with Bonferroni correction, which leads to an overall level of significance of 0.05 (one-sided)—a standard choice in this type of design.

**Figure 1:**
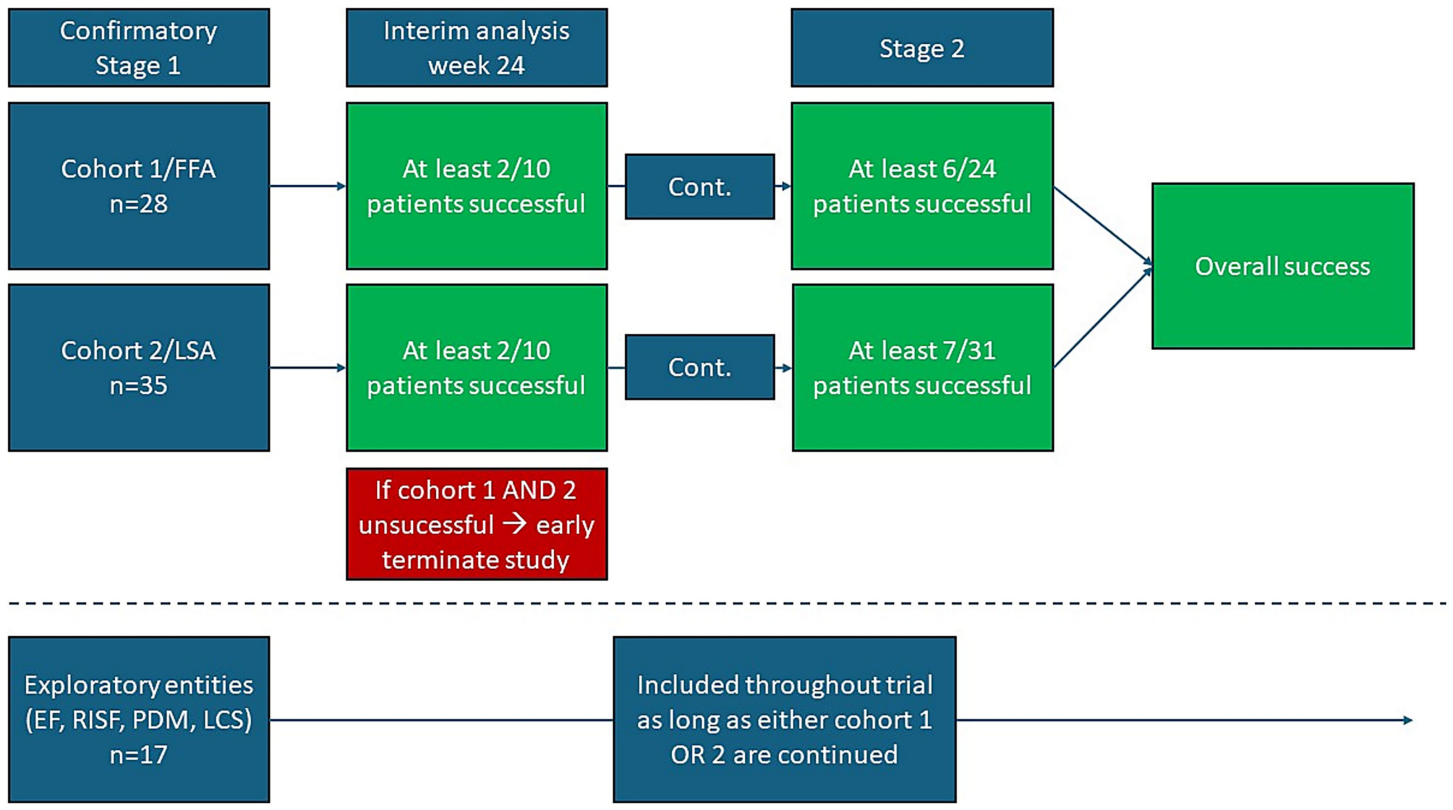
Illustration of the proposed two-stage Simon design. In stage 1, at least 2 out of 10 included patients should be successful in either cohort 1/FFA or cohort 2/LSA. In stage 2, at least 6 out of 24 included patients should be successful in either cohort 1/FFA or 7 out of 31 in cohort 2/LSA to achieve statistical significance. The exploratory entities (EF, RISF, PDM, and LCS) may be recruited throughout the trial. Their success rate does not contribute to the statistical significance of the entire design. EF, eosinophilic fasciitis; FFA, frontal fibrosing alopecia; LCS, linear circumscript scleroderma; LSA, lichen sclerosus et atrophicus; PDM, pansclerotic disabling morphea; RISF, radiation-induced skin fibrosis.

The two-stage Simon design is a statistical method used in phase 2 clinical trials to determine whether a treatment has sufficient activity to warrant further study. The design involves two stages: in the first stage, a small number of patients are treated, and if the treatment shows promise, the trial proceeds to the second stage with additional patients. This approach helps to minimize the number of patients exposed to potentially ineffective treatments while ensuring that promising treatments are identified early. It is based on two response probabilities: the response probability of a poor drug, p_0_, and that of a good drug, p_1_. In our trial, p_0_ was set to 0.10, and p_1_ was set to 0.33 in both subcohorts. Assuming p_0_, the trial should be successful with a probability of 5% only (one-sided level of significance), and assuming p_1_, the trial should be successful with a probability of 80% in power subcohort 1/FFA or 85% in power subcohort 2/LSA. The different values of the power were chosen due to the feasibility of patient recruitment. Since we apply this design in two cohorts (FFA and LSA), and we want to control the overall type 1 error, we choose a level of significance of 0.025 (one-sided) in each cohort. In cohort 1/FFA, 24 patients will be included, and in cohort 2/LSA, 31 patients will be included. An interim analysis after 10 patients should reveal at least two successes in each cohort. Otherwise, the respective cohort is stopped for futility. After a total of 24 patients, at least 6 successes have to be observed to be successful in cohort 1/FFA, and after 31 patients in cohort 2/LSA, 7 successes have to be observed to be successful in this cohort. To account for potential dropouts, which are common in this type of design, 28 patients will be planned in subcohort 1/FFA and 35 in subcohort 2/LSA. However, recruitment of patients will go on in both subcohorts until the number of evaluable patients is reached. Thus, depending on the number of dropouts, the sample size might be modified in both subcohorts during the trial. This may not be an adaptation in the biometrical sense. In theory, a cohort might be stopped when six successes have been reached with fewer than 24 recruited patients (cohort 1/FFA) or 7 patients with fewer than 31 recruited patients (cohort 2/LSA). However, we decided not to use this option, as we want to gather information about safety and secondary endpoints in both cohorts as well as in the exploratory cohorts (EF, RISF, PDM, and LCS). It is not unethical to include the planned number of patients, as it is intended to repurpose drugs licensed in other disease entities. The two-stage Simon design is widely used due to its efficiency and ethical considerations in clinical research (11). Figure 1 illustrates the design.

The sample size of *N* = 80 is calculated based on the assumption of uneven recruitment between the entities, allowing a sample size of *n* = 4 for the rarest diseases and up to *n* = 10 for rare entities with higher recruitment, and based on the sample size necessary to pass the interim analysis of the two-stage design in the more common entities (LSA, FFA). Thus, disease entities with very low incidence (RISF, EF, PDM, and LCS) are recruited as exploratory groups, while entities with higher incidence (LSA and FFA) are employed as main study groups (Table 2).

**Table 2:** Disease entities and allocated patients.

Disease	Allocated patients (*N* = 80)
Extragenital and genital Lichen sclerosus et atrophicus (LSA) in adolescents and adults	*n* = 35 (43.8%)
Frontal fibrosing alopecia (FFA)	*n* = 28 (35%)
Eosinophilic fasciitis (EF)	As many as possible, up to *n* = 17 (21.3%)
Radiation-induced skin fibrosis (RISF)	
Pansclerotic disabling morphea (PDM)	
Linear circumscript sclerodermia (LCS)	

Meaningful response criteria have been established for some fibrotic skin diseases (Localized Scleroderma Cutaneous Assessment Tool (LoSCAT), Modified Localized Scleroderma Skin Severity Index (mLoSSi), Localized Scleroderma Damage Index (LoSDI) for localized scleroderma/morphea; Frontal Fibrosing Alopecia Severity Index (FFASI) and FFASI-Quality of Life (QoL) for FFA; and Lichen Sclerosus Clinical Index of Severity (LSCIS) and CIV for LSA in adults). Other fibrotic diseases lack validated scoring systems. Due to the level of fibrosis, no single scoring system can be applied to all entities studied. Therefore, improvement of IGA will be used as the primary endpoint to allow for a common endpoint.

Typically, a target IGA of 0 or 1 is used in studies of atopic dermatitis or psoriasis. However, in fibrotic diseases, restitutio ad integrum is unlikely because of already occurred damage. Therefore, an IGA improvement (ΔIGA) of 1 point seems much more feasible. At the interim analysis, an improvement of 1 point on the IGA will allow us to proceed to the second stage of the study.

Other global scores will also be applied, but refer to patient-oriented outcomes, such as DLQI, Itch-and Pain-NRS, or EuroQol five dimensions (EQ-5D-QoL), and will be studied as secondary endpoints, as well as disease-specific response criteria. While the diversity of entities under investigation is addressed by disease-specific scores and imaging techniques, clinical outcomes are also captured using common standardized scoring systems and imaging techniques, allowing common primary endpoints (Table 1).

We propose a 52-week treatment period, due to the slow nature of progression as well as treatment response in fibrotic skin diseases. As this is a phase 2b trial in chronic progressive diseases with the possibility of irreversible damage and without licensed therapies of high efficacy, no placebo group will be included. Disease-specific rescue therapies are allowed for disease progression. All patients will be followed through week 52.

The treatment phase for each patient will continue until week 52 with a follow-up visit at week 56. The planned secondary endpoint at week 52 is a 2-point improvement on the IGA scale.

Our project will generate further endpoints from response criteria, as suggested by Vandevanter et al. (12).

Signaling pathways and cellular responses involved in fibrosis will be investigated in blood and tissue samples using bulk and single-cell RNA sequencing, proteomics, and immunohistochemistry, aiming to better understand the pathophysiology within the entities, as well as to allow a subgroup analysis of responders after the trial.

### 2.3 Key inclusion criteria

The study’s inclusion criteria are as follows:

- Patients suffering from LSA, FFA, EF, RISF, PDM, and LCS:
- Disease manifestation within a maximum of 12 months prior to baseline.
- Active disease 3 months prior to screening.
- Age 12 years or older (depending on the in-label status of the chosen therapy).

The primary endpoint at week 24 is defined as follows:

- Clinical improvement with a change of Investigator Global Assessment (IGA) of at least 1 point from baseline (Table 3).

**Table 3:** Comparison of disease-specific Investigator Global Assessment (IGA) grades for investigated entities.

IGA	LSA	FFA	EF	RISF
0 = Clear (no disease activity)	No inflammation, disease activity, or discomfort.	No perifollicular erythema, scaling, or further hairline recession. No evidence of inflammation.	Clear	Clear
1 = Minimal (minimal activity)	Very slight changes in skin texture or color, with barely noticeable white patches. There may be mild itching or discomfort, but no significant impact on daily activities.	Minimal perifollicular scaling; no significant progression of hairline recession.	Minimal skin thickening or hardening with barely noticeable swelling. Slight “orange peel” appearance only visible upon close inspection. No restriction in movement. Minimal discomfort that does not affect daily activities.	Mild erythema and/or slight fibrosis
2 = Mild (low disease activity)	More noticeable white patches and slight thickening of the skin. Itching and discomfort are more frequent, but still manageable. There might be some minor fissures or cracks in the skin.	Mild perifollicular faint erythema and/or mild perifollicular scaling, slight progression of recession (<1 cm in 6 months).	Noticeable skin induration and mild swelling in limited areas. “Orange peel” appearance is visible without stretching the skin. Slight restriction in the movement of affected areas. Occasional pain or discomfort that is manageable. Minor impact on some activities.	Moderate erythema, fibrosing tissue with shrinking, slight pain, or pruritus
3 = Moderate (active disease)	White patches are more extensive, and the skin is significantly thickened. Itching and discomfort are persistent and can interfere with daily activities. There may be more pronounced fissures, cracks, and possibly some bleeding.	Noticeable perifollicular erythema, scaling, and recession (1–2 cm in 6 months).	Significant skin induration, hardening, and swelling in multiple areas. Pronounced “orange peel” texture and possible hyperpigmentation. Moderate restriction in joint movement. Frequent pain and discomfort that interferes with daily activities. Some difficulty with the range of motion.	Severe erythema, fibrosis with initial shrinking, or/and moderate pain
4 = Severe (high disease activity/progression)	Extensive white patches with severe thickening and hardening of the skin. Itching and pain are constant and can be debilitating. Deep fissures, cracks, and bleeding, and the condition may significantly impact the quality of life and daily functioning.	Marked perifollicular erythema, scaling, and significant progression (>2 cm in 6 months); possible eyebrow/sideburn loss.	Extensive skin induration and hardening with marked swelling. Prominent “orange peel” appearance and hyperpigmentation. Severe restriction in movement with joint contractures. Persistent pain that significantly impacts quality of life and daily functioning. May have systemic symptoms (fever, marked weight loss, debilitating fatigue, and malaise).	Swelling and severe dark red erythema or/and severe disfiguring fibrosis, ulceration, extensive shrinking, or/and severe pain

The secondary endpoints at week 52 encompass the following outcomes:

- Clinical improvement with a change of Investigator Global Assessment (IGA) of at least two points from baseline.
- Improvement of quality of life as a reduction of 50% or more in Dermatological Life Quality Index (DLQI) from baseline.
- Itch improvement of at least 4 points on a 10-point Numeric Rating Scale.
- Skin pain improvement of at least 4 points on a 10-point Numeric Rating Scale.
- A composite endpoint consisting of the following objective and subjective symptoms:
- Improvement of IGA ≥ 1 point.
- Itch improvement of ≥ 2 points on a 10-point NRS OR pain improvement of ≥ 2 points on a 10-point NRS.
- Clinical improvement in disease-specific scores (Table 1).
- Quality of life improvement measured by the EuroQol Group Quality of Life Five-Dimension Youth-Five-Level Questionnaire (EQ-5D-Y-5 L) in children and adolescents, and the EuroQol Group Quality of Life Five-Dimension Five-Level Questionnaire (EQ-5D-5L) in adults.

This study is conducted in accordance with the German Medicines Act. To enable recruitment of a sufficient number of patients with rare diseases, we propose a multicenter study with a lead center to organize and coordinate the investigator-initiated trial via its center for clinical studies. All centers may include all selected diseases, since most are very rare and a single-center approach is not feasible. For every included disease, expert principal investigators (PIs) at selected centers should be in charge of one specific disease for developing specific parts of the protocol. The central center for clinical studies will provide guidance on the cost estimate for submission, project management, monitoring of all centers, as well as pharmacovigilance.

## 3 Discussion

Despite the rarity of debilitating fibrotic skin disease, therapeutic nihilism is unjustified. Repurposing already approved drugs for the treatment of rare diseases is a promising strategy that leverages existing medications to address unmet medical needs. This approach not only accelerates the availability of treatments for patients with rare and debilitating conditions but may also provide valuable insights into the underlying pathophysiology of these diseases. Additionally, understanding the pathophysiology of rare diseases can inform the design of clinical trials and improve patient outcomes. Furthermore, since these drugs have already been approved for other indications, their safety profiles are well-established, reducing the risk of adverse effects.

The rarity of the conditions hinders clinical trials in fibrotic diseases. This proposal outlines a novel approach to studying a drug in this setting, establishing a basket trial to evaluate the efficacy and safety of a common therapy in LSA, EF, FFA, RISF, PDM, and LCS. Successful implementation of this strategy could significantly improve outcomes for patients suffering from these debilitating conditions. For example, by targeting a key cytokine involved in the fibrotic process at an early stage, this strategy has the potential to halt or even reverse disease progression if treatment is initiated early enough.

The suggested approach combines rare and slightly less rare diseases in a clinical trial by identifying common endpoints. This can increase the statistical power of the study and make it easier to demonstrate the efficacy of repurposed drugs. If the different fibrotic conditions share a common pathophysiological pathway, they can be studied together to assess the impact of a treatment on all conditions. This not only accelerates the development of new therapies but also broadens the scope of research, benefiting a larger patient population.

Generating statistical endpoints for a basket trial without a hard endpoint is challenging due to the variability and heterogeneity of the diseases involved (LSA, FFA, RISF, EF, PDM, and LCS). Without a clear, measurable outcome, it becomes difficult to assess the efficacy of treatments across different patient groups. Therefore, we propose using common endpoints such as IGA and QoL assessments (DLQI and EQ-5D) as composite measures for the primary endpoint. These endpoints are necessary because, in fibrotic diseases, restitutio ad integrum is not probable because of the already existing fibrosis. Only a reduction of fibrosis or even just the stopping of progression is probable, as has been demonstrated in SSc, where the modified Rodnan Skin Score (mRSS) was reduced but never reached complete remission (13).

This limitation can certainly introduce variability and reduce the study’s statistical power. To counteract this, disease-specific hard endpoints (i.e., LSCIS, FFASI, LoSCAT, etc.) will be used as secondary outcome measures.

Possible pitfalls include a missing link between the pathophysiology of these diseases. This will be addressed by preclinical studies on archival tissue samples to establish an IL-13 signature as a common treatable target.

Our approach has certain advantages: the adapted two-stage Simon Design provides us the flexibility to address rare diseases such as EF, RISF, PDM, and LCS by utilizing the statistical power of more common but undertreated diseases like LSA and FFA.

It therefore serves undertreated patient populations who would probably never be included in clinical trials. To counteract the ever-rising research and development (R&D) costs and the risk of failure of clinical development, we designed the study to be multicenter but uninational, with a population of *N* = 80 patients, under the premise “Quality beats quantity” (14).

## Data Availability

The original contributions presented in the study are included in the article/supplementary material, further inquiries can be directed to the corresponding author.
